# Mother's Characteristics and Socioeconomic Status as Possible Risk Factors for Children's Caries in Jordan

**DOI:** 10.1155/2022/2006088

**Published:** 2022-03-16

**Authors:** Omar Al-Rashdan, Zaid AlZoubi, Mahmoud Ibrahimi, Amal Al-khraisha, Nabeel Almajali

**Affiliations:** ^1^Department of Oral and Maxillofacial Surgery, Jordanian Royal Medical Services, Amman 11855, Jordan; ^2^Conservative Dentistry Department, Jordanian Royal Medical Services, Amman 11855, Jordan; ^3^Pediatric Dentistry Department, Jordanian Royal Medical Services, Amman 11855, Jordan

## Abstract

**Background:**

Clinical oral health status of children affects their health-related quality of life. A major determinant of oral health is early childhood caries, which possesses a negative effect.

**Objective:**

The primary objective of the study was to examine the association between socioeconomic status and different mother's characteristics and the risk of dental caries in children using the decay missing filled (DMF) score as an indicator.

**Methods:**

This was a cross-sectional descriptive study that was in the pediatric dental clinic in Hashem Ibn Al-Hussein medical military hospital in Jordan. Pearson correlation was used to examine associations between two continuous variables. Linear regression was used to detect variables that might predict the decayed missing filled teeth (dmft) score of the child.

**Results:**

A total of 264 children were enrolled in the study. Average age of children was 4.80 ± 1.99 years, and average mothers' age was 32.74 ± 5.68. Mothers had an average DMFT score of 8.84 ± 5.39, while children had an average dmft score of 6.17 ± 4.82. There was no association between the mother's age and the dmft score of the child (Pearson correlation = 0.08, and *P* value = 0.215). However, a moderate, statistically significant correlation was found between the mother's and the child's DMFT scores (Pearson correlation = 0.418, and *P* value = 0.001). Children who belonged to low and middle-income families had a higher dmft score compared to those that belonged to families with high income.

**Conclusion:**

Socioeconomic status of the family, mother's habits, dental hygiene, and education level are important factors that influence the child's oral health. Strategies that focus on children with these characteristics can help them achieve better oral health.

## 1. Introduction

Dental caries is the most common disease in children [1]. Clinical oral health status of children affects health-related quality of life [2]. In children aged 2–5 years, early childhood caries (ECC) had a negative impact on oral health-related quality of life. As the severity of ECC increased, so did the negative effect on the quality of life of the child [3]. The effect of dental caries is not confined to the quality of life but extends to other aspects of children's health. Acs et al. showed that 13.7% of children with ECC weighed less than 80% of their ideal weight. Following dental rehabilitation, these children displayed statistically significant enhanced growth rates [4]. In addition, severe dental decay in children may contribute to failure to thrive (FTT) [5]. Identifying high-risk children to prevent early childhood caries is cost-effective [6].

In view of the abundant evidence that supports the important consequences of dental caries on the children's medical, psychological, and emotional status, it is crucial to identify children at high risk for dental caries and start early management. Many factors lead to increased dental caries in children, and these include familial socioeconomic status, inadequate parental education, and reduced access to dental care [7]. Mothers are the primary care providers for children, and many studies showed that the characteristics and behavior of mothers had a significant effect on the prevalence of their children's dental caries. De Souza et al. conducted a study in Brazil that included 77 mother-child pairs with ages ranging from 12 to 36 months. They revealed that caries was 22.5 times higher in children with mothers who had decayed teeth [8]. Alade et al.'s study that was conducted in Nigeria examined the association between several maternal factors and dental caries in children. Findings revealed that children whose mothers had caries were six times more likely to have early childhood caries than children with mothers who had no caries [9]. In Korea, there was a positive correlation between the mothers' and children's decayed, missing, and filled teeth (DMFT) scores. Additionally, children from families with a low income had higher decayed teeth score [10]. Gokhale and Nuvvula examined the correlation between the socioeconomic and working status of the parents and dental caries in children. They revealed that those who belonged to a family of both working parents or of low socioeconomic status were more likely to have dental caries [11].

Another factor that plays an important role in oral health is the parenting styles of parents or how parents raise their children. Children who were raised by permissive parents had a higher dmft score than those raised by authoritative parents [12]. In addition, children with parents who were not involved in their children's oral health had a higher dmft score [13].

In Jordan, BaniHani et al. assessed maternal knowledge of risk factors for early childhood caries. Unfortunately, the study revealed that most mothers had poor knowledge of their children's oral health [14]. To our knowledge, no study was conducted in Jordan to examine the relationship between maternal characteristics and dental caries in children. Our study sheds light on these risk factors and helps identify children who need early screening. The financial resources in developing countries are limited, and the provision of dental health is an enormous challenge. Therefore, prevention of early childhood caries and starting healthy practices at an early age set the stage for continuous implementation of good dental health from early childhood, through adolescence, and reaching adulthood.

The aim of this study was to examine maternal factors associated with increased risk of dental caries in children and assess the prevalence and severity of dental caries in children who attended the dentistry clinic at a tertiary hospital.

## 2. Methodology

This was a cross-sectional descriptive study that was conducted between March and June 2021 in the pediatric dental clinic in Hashem Ibn Al-Hussein medical military hospital in Jordan. Inclusion criteria were as follows: all children visiting the pediatric dental clinic who co-operated during the oral examination were possible candidates for enrollment in the study. The mothers (regardless of their age) were approached for consent to participation representing themselves and the children (as their legal guardians). Exclusion criteria were as follows: children with disabilities that may affect oral hygiene practices were excluded and children who did not cooperate during the dental clinical examination process.

Prior approval from the Institutional Ethical Committee was obtained before launching the study. A signed consent from the mothers of the children (guardians) was obtained before approval to conduct the interviews and dental examination.

A data collection form was constructed to gather relevant information. The form was developed after an extensive review of the literature and was revised by two external experts. A pilot study of 10 participants (who were not included in the final analysis) was conducted to evaluate the time needed for the interview and the appropriateness of the data collection form. Clinical data acquisition involved clinical examination of both the mother and the child. The clinical examination was conducted by two dentists for each patient to confirm the results, and conflict was resolved by consulting a pediatric or a conservative dentist. The clinical examination was carried out by mirror number 5 and 23/17 dental explorer. The DMFT scores were calculated based on the consensus results obtained from the clinical examination. Mothers were assessed for decayed, missing, and filled teeth in the permanent dentition (DMFT index), and their children were assessed for decayed, missing, and filled teeth in the deciduous dentition (dmft index).

Demographic and socioeconomic data were collected through conducting interviews with the mother and included age, monthly income, mother's educational level, and work status. All the relevant data were documented in the data collection form and entered to the statistical software.

### 2.1. Statistical Analysis

Continuous data were presented as mean ± SD, and categorical data were presented as frequency (%). Pearson correlation was used to examine associations between two continuous variables. Linear regression was used to detect variables that might be predictors of the DMFT score. *P* values of less than 0.05 were considered statistically significant. The software package SPSS® (IBM, USA) version 25 was used for analysis.

## 3. Results

A total of 264 children were enrolled in the study. Average age of children was 4.80 ± 1.99 years (maximum age 15 years, minimum age 1 year). Average mothers' age was 32.74 ± 5.68 years (maximum age 50 years and minimum age 20 years). The general characteristics of the participants are shown in Table 1. The average dmft score for the children was 6.17 ± 4.82, only 38 children (14.9%) had a dmft score of zero, the lowest dmft score was one (9 children, 3.5%), and the highest was twenty (2 children, 0.8%). The average DMFT score for the mothers was 8.84 ± 5.39, only seven participants had a DMFT score of zero (2.8%), the lowest DMFT score was one (11women, 4.4%), and the highest was twenty-five (1woman, 0.4%).

Families had an average number of children of 3.37 ± 1.68, with an average number of females 1.67 ± 1.28, and 1.71 ± 1.19 males. Most of the visits to the dentist clinic were not regular follow-ups but rather emergency visits 193 (77.5%).

Several correlations between the mother's/family characteristics and the child's dmft score were examined. Mothers' age and the number of children in the family were not associated with the dmft score. On the other hand, the mother's DMFT score was significantly associated with the child's dmft score, Pearson correlation = 0.418, *P* value = 0.001, as shown in Table 2.

The most common method of cleaning teeth in the children was the toothbrush, and one hundred and sixty-one children (61.0%) cleaned their teeth with a toothbrush, as shown in Table 3. This method was also the most common one for cleaning teeth by the mothers, as shown in Table 4.

Possible predictors of the child's dmft score were assessed using univariate and multivariate linear regression, as shown in Table 5. The multivariate linear regression was performed using backward analysis, and *P* value of the total model was 0.013. Results showed that the only variable that was statistically significant was family monthly income. Children who belonged to families with a monthly income of less than 500–1000 (JOD) or lower than 500 (JOD) had a higher dmft score compared to those who belonged to families with a monthly income of >1000 (JOD).

## 4. Discussion

Most of the enrolled children were preschoolers (average 4.8 years) and the percentage of boys in the study was slightly higher than girls. The average dmft score identified in our study was comparable to that revealed in a recent study that was conducted by Bani Hani et al. in Jordan, 6.04 ± 1.2 [14]. However, it was higher than that detected in regional and neighboring countries. In northern Palestine, the average dmft score was 2.46, and the percentage of caries-free children was 24% [15]. This can be explained by the fact that the study in Palestine included children between 4 and 5 years, while our study had a higher age range for inclusion. As the age increases through adolescence, dmft scores increase [16]. Moreover, the DMFT score of the mothers in our study was also higher than in other countries [10]. The percentage of caries-free children in our study was also lower than that in developed countries, and 69% of children at the age of three in Sweden were caries free [17].

Most of the mothers were unemployed and did not have high education level. In addition, two thirds of the enrolled children belonged to families with low monthly income. This is expected since the medical military services provide medical care to soldiers and their families with an average income lower than 500 Jordanian dinars per month.

This high increase in the percentage of mothers with low education level and low family income might explain the increased average of dmft score in children enrolled in our study since these two factors were predictors of high dmft scores in children.

Most of the visits were for emergency causes, and this trend was discovered in other studies where 25% of the pediatric visits to the dental clinic were for emergencies [18]. This finding implies that parents are not well educated in terms of the importance of early dental care and their effect on the occurrence of dental caries [19].

The lack of association between the number of children in the family and dmft score is not consistent with other studies that found that a higher number of siblings might be correlated with higher dental caries [20, 21].

The DMFT score of the mothers was significantly correlated with the dmft score of the children. The effect of mothers' oral health on their children's dmft scores and risk for dental caries were well documented in many studies [10, 22, 23]. Therefore, intensive counseling of the child and the mother as the primary caregiver was one of the most important strategies suggested to reduce the risk for caries in children [24].

The most common method used to maintain dental hygiene was the tooth brush which is the most effective method regardless of the type [25]. The only significant predictor of dmft scores of the children was monthly income. Although the level of education of the mother neared statistical significance, children whose mothers had elementary education had significantly higher dmft score compared to those whose mothers had a university education, 7.22 and 5.19, respectively, *P* value = 0.061. Other studies have shown the link between mothers' low education level and increased risk for dental caries in their children [26].

Children who belonged to families in the two monthly income groups less than 1000 JOD (<500, 500–1000 JOD/month) had significantly higher dmft scores (6.38, *P* value = 0.043 and 6.39, *P* value = 0.019, respectively) compared to those that belonged to the high-income group of more than 1000 JOD/month. This result is in concordance with findings from different parts of the world [23, 27, 28]. Bani Hani et al. assessed the association between maternal knowledge and childhood caries in Jordan, and low family income constituted a significant barrier to seek dental treatment [14].

Our study had certain limitations among which are low sample sizes. Moreover, the participants were enrolled from one hospital, and a multicentered study would have been more representative and resulted in a larger sample size. Another limitation is the use of the DMFT index to evaluate dental caries, and this method does have its drawbacks when used for dental caries data analysis [29].

## 5. Conclusion

Dental caries in children exerts emotional, physiological, and psychological effects. In our study, the most common method of dental hygiene was the toothbrush. Providing education on the best way to use the toothbrush is reasonable. In addition, other methods of dental hygiene such as dental floss and mouth gargles should be promoted. Mothers with high DMFT scores, mothers with modest education levels, and families with low monthly income were predictors of high dmft scores in children. Therefore, children who belong to these families and environments should be identified, and appropriate education, motivation, and support should be directed towards them to improve their dental health.

## Figures and Tables

**Table 1:** General characteristics of the children's families.

Variable	Frequency (%)
Gender	
Male	148 (56.1%)
Female	116 (43.9%)
Education of the mother	
Elementary	32 (12.2%)
Secondary	111 (42.0%)
University or higher	98 (37.1%)
Not specified	23 (8.7%)
Family monthly income	
<500 dinars per month	195 (73.9%)
500–1000 dinars per month	54 (20.4%)
>1000 dinars per month	11 (4.2%)
Not specified	4 (1.5%)
Education of the father	
Elementary	15 (5.7%)
Secondary	60 (22.7%)
University or higher	18 (6.8%)
Not specified	171 (64.8%)
Does the mother work or not	
Mother is not employed	206 (78.0%)
Mother is employed	51 (19.3%)
Not specified	7 (2.7%)
Mother's working hours^*∗*^	
<8 hours per day	6 (11.8%)
8 hours per day	44 (86.3%)
>8 hours per day	1 (2.0%)

*N* = 264.^*∗*^, total number of employed mothers was 51.

**Table 2:** Pearson correlation of different variables with dmft score of the children.

Variable	Pearson correlation	*P* value
Mother's age	0.08	0.215
Number of children in the family	0.057	0.336
Mother's DMFT score	0.418	0.001

**Table 3:** Methods of dental hygiene practiced by the children.

Cleans by toothbrush	
Yes	161/264 (61%)
No	103/264 (39%)
Frequency	
Daily	99/161 (61.5%)
1 time	62 (62.6%)
2 times	26 (26.3%)
3 times	6 (6.0%)
Not specified	5 (5.1)
Weekly	60/161 (37.3%)
1 time	18 (30.0%)
2 times	22 (36.6%)
3 times	15 (25.0%)
4 times	1 (1.7%)
5 times	1 (1.7%)
Not specified	3 (5.0%)
Monthly	2/161 (1.2%)
2 times	2 (100%)
Cleans by dental floss	
Yes	3/264 (1.1%)
No	261/264 (98.9%)
Frequency	
Daily	3/3 (100%)
1 time	3 (100%)
Cleans by gargle	
Yes	8/264 (3%)
No	256/264 (97%)
Frequency	
Daily	6/8 (75.0%)
1 time	2 (33.3%)
2 times	3 (50%0
Not specified	1 (16.7%)
Weekly	1/8 (12.5%)
1 time	1 (100%)
Monthly	1/8 (12.5%)
1 time	1 (100%)

**Table 4:** Methods of dental hygiene practiced by the mothers.

Cleans by toothbrush	
Yes	241/264 (91.3%)
No	23/264 (8.7%)
Frequency was not determined	2/241 (0.8%)
Frequency determined (daily, weekly, monthly)	239/241 (99.2%)
Frequency	
Daily	175/239 (73.2%)
1 time	75 (42.9%)
2 times	75 (42.9%)
3 times	16 (9.1%)
4 times	1 (0.6%)
Not specified	8 (4.5%)
Weekly	61/239 (25.6%)
1 time	8 (13.1%)
2 times	26 (42.6%)
3 times	22 (36.1%)
5 times	4 (6.6%)
Not specified	1 (1.6%)
Monthly	3/239 (1.2%)
1 time	2 (66.7%)
2 times	1 (33.3%)
Cleans by dental floss	
Yes	14/264 (5.3%)
No	250/264 (94.7%)
Frequency	
Daily	10/14 (71.4%)
1 time	5 (50.0%)
2 times	3 (30.0%)
3 times	2 (20.0%)
Weekly	3/14 (21.4%)
1 time	2 (66.7%)
2 times	1 (33.3%)
Monthly	1/14 (7.2%)
1 time	1 (100%)
Cleans by gargle	
Yes	50/264 (18.9%)
No	214/264 (81.1%)
Frequency	
Daily	21/50 (42.0%)
1 time	11 (52.4%)
2 times	5 (23.8%)
3 times	2 (9.5%)
Not specified	3 (14.3%)
Weekly	19/50 (38.0%)
1 time	12 (63.1%)
3 times	4 (21.1%)
Not specified	3 (15.8%)
Monthly	10/50 (20.0%)
1 time	7 (70.0%)
2 times	2 (20.0%)
3 times	1 (10.0%)

**Table 5:** Linear regression analysis of possible predictors of the DMF scores of the children.

Independent variables	Univariate linear regression			Multivariate linear regression			DMF score ± SD
	*B*	95% CI	*P* value	*B*	95% CI	*P* value	
Education of the mother			0.027^*∗*^				
Elementary (reference)							7.22 ± 5.29
Secondary	−0.838	−2.399–0.722	0.291^*∗*^				6.45 ± 4.36
University or higher	−2.097	−3.695–0.500	0.010^*∗*^	−1.293	−2.646–0.060	0.061	5.19 ± 4.77
Family monthly income			0.023^*∗*^				
>1000 Jordanian dinars/month (reference)							1.64 ± 2.34
500–1000 Jordanian dinars/month	3.529	0.753–6.305	0.013^*∗*^	3.268	0.550–5.987	0.019^*∗*^	6.39 ± 4.49
<500 Jordanian dinars/month	3.510	0.994–6.026	0.006^*∗*^	2.657	0.085–5.229	0.043^*∗*^	6.38 ± 4.87
Does the mother work			0.131				
Does not work (reference)							6.26 ± 4.74
Employed	−1.140	−2.621–0.341	0.131				5.12 ± 4.61

*P* value of the multivariate model was 0.004. ^*∗*^Statistically significant. CI: confidence interval. *B* is the regression coefficient, the larger the value, the higher the effect. Positive value of *B* suggests an increase in DMF score, and negative value suggests a decrease in the DMF score of the child.

## Data Availability

Data can be available upon request and in a very restricted manner to coincide with the Jordanian Royal Medical Services ethics on the confidentiality of the patient's information.
